# Wound-Healing Effects of Mesenchymal Stromal Cell Secretome in the Cornea and the Role of Exosomes

**DOI:** 10.3390/pharmaceutics15051486

**Published:** 2023-05-13

**Authors:** Seungwon An, Khandaker Anwar, Mohammadjavad Ashraf, Hyungjo Lee, Rebecca Jung, Raghuram Koganti, Mahmood Ghassemi, Ali R. Djalilian

**Affiliations:** 1 Department of Ophthalmology and Visual Sciences, University of Illinois at Chicago, Chicago, IL 60612, USA; kanwar@uic.edu (K.A.); mashra5@uic.edu (M.A.); hjolee1@uic.edu (H.L.); rjung6@uic.edu (R.J.); rkogan3@uic.edu (R.K.); ghassemi@uic.edu (M.G.); 2 Department of Pathology, Shiraz University of Medical Sciences, Shiraz 71348-14336, Iran

**Keywords:** mesenchymal stromal cells, mesenchymal stem cell-conditioned media, exosomes, reconstituted–conditioned media, wound healing

## Abstract

Mesenchymal stromal/stem cells (MSCs) and their secreted factors have been shown to have immunomodulatory and regenerative effects. In this study, we investigated human bone-marrow-derived MSC secretome (MSC-S) for the treatment of corneal epithelial wounds. Specifically, we evaluated the role of MSC extracellular vesicles (EV)/exosomes in mediating the wound-healing effects of the MSC-S. In vitro studies using human corneal epithelial cells showed that MSC-CM increased cell proliferation in HCEC and HCLE cells, while EV-depleted MSC-CM showed lower cell proliferation in both cell lines compared to the MSC-CM group. In vitro and in vivo experiments revealed that 1X MSC-S consistently promoted wound healing more effectively than 0.5X MSC-S, and MSC-CM promoted wound healing in a dose-dependent manner, while exosome deprivation delayed wound healing. We further evaluated the incubation period of MSC-CM on corneal wound healing and showed that MSC-S collected for 72 h is more effective than MSC-S collected for 48 h. Finally, we evaluated the stability of MSC-S under different storage conditions and found that after one cycle of freeze–thawing, MSC-S is stable at 4 °C for up to 4 weeks. Collectively, we identified the following: (i) MSC-EV/Exo as the active ingredient in MSC-S that mediates the wound-healing effects in the corneal epithelium, providing a measure to optimize its dosing for a potential clinical product; (ii) Treatment with EV/Exo-containing MSC-S resulted in an improved corneal barrier and decreased corneal haze/edema relative to EV/Exo-depleted MSC-S; (iii) The stability of MSC-CM for up to 4 weeks showed that the regular storage condition did not significantly impact its stability and therapeutic functions.

## 1. Introduction

Mesenchymal stem cells (MSCs) are adult stem cells characterized by specific cell surface markers and their ability to differentiate into bone, cartilage, and fat cells [1,2,3,4,5]. MSCs are found in many tissues throughout the body, including bone marrow, adipose tissue, and umbilical cord tissue [1,2]. MSCs possess the ability to self-renew and, thus, maintain a population of cells that can be used for tissue regeneration and wound healing [6]. MSCs have been studied in a variety of niches including immunomodulatory [7,8,9], regenerative [10,11,12], anti-inflammatory [13], pro- and anti-angiogenic, neuro-protective, and organ-healing effects [14,15].

The therapeutic effects of MSCs are mediated predominantly via paracrine factors involving proteins, RNA, and exosomes/extracellular vesicles [16,17,18,19,20,21]. The MSC secretome, a diverse collection of bioactive mediators that is secreted into the cellular microenvironment, exerts a powerful influence on neighboring tissues and can modify their phenotype [22]. MSCs can inhibit the activation of pro-inflammatory Th1 and Th17 cells while promoting the activity of Treg cells, the overall effect of which can be leveraged to reduce graft rejection [23,24]. Similar immunosuppressive properties have been demonstrated when B cells and dendritic cells are exposed to the MSC secretome [25,26]. Additionally, MSCs have been shown to support or restrict angiogenesis in different models which further highlights their flexibility as a therapeutic modality [27,28].

While MSCs are a promising form of therapy and lack immunogenicity, there are still risks in their utilization in therapies. Adverse effects of MSC therapy in clinical trials include thromboembolism and fibrosis, and there is some evidence that suggests that MSCs may influence tumorigenesis [29,30]. Thus, there is a need to harness the beneficial effects of MSC therapy while minimizing the risk of adverse effects. One approach that has gained popularity in recent years is to use media containing the MSC secretomes but lacking the MSCs themselves. Often referred to as MSC-conditioned media (MSC-CM), this approach can recapitulate many of the therapeutic effects of cell therapy with fewer side effects. Studies have shown that MSC-CM can promote the repair of chemical and mechanical injuries [3,31,32,33]. Furthermore, cell-free therapies can be manufactured in higher volumes with lower production costs as well [34]. MSC-CM has been shown to promote fibroblast and endothelial cell migration, accelerating the wound-healing process [35]. One systematic review reported that topical MSC-CM has been shown to improve wound closure and vascularization across a multitude of studies compared to control treatment in vivo models [36]. These results were true for wounds with origins ranging from diabetes, psoriasis, burns, and radiation [36]. MSC-CM has also been shown to promote neurite regeneration, restrict inflammation, and reduce scar formation in the wound-healing process [37,38,39]. The recency and robustness of this body of work highlight MSC-CM as an effective alternative to traditional MSC therapy in supporting wound healing and the treatment of various diseases.

Exosomes (Exo), or more generally extracellular vesicles (EVs), have likewise been identified as one of the principal components that mediate the effects of MSC-based therapies [40]. The efficacy of MSC-EV or MSC-Exo has been reproduced in many studies across multiple tissue models. MSC-EV/Exo has been shown to promote hepatic regeneration while reducing fibrosis in models of liver disease [41,42,43]. They have also demonstrated efficacy in ameliorating inflammation and increasing survival in models of kidney injury [44,45]. MSC-EV/Exo has also improved outcomes in preclinical studies of myocardial infarction, stroke, and pulmonary hypertension [46,47]. Interestingly, studies using MSC-EV/Exo as a vehicle for the delivery of microRNAs have reported beneficial effects on increasing neural plasticity as well [48,49]. Thus, delivering exosomes and extracellular vesicles may constitute an even greater targeted therapy than media conditioned by MSCs or introducing MSCs directly. Although the mechanism of the in vivo effects of exogenously administered exosomes or the targeted delivery of exosome is not fully elucidated, MSC-EV/Exo is being considered as a new cell-free therapeutic paradigm for clinical translation [50,51,52,53].

While the literature on MSC-EV/Exo therapies is rapidly expanding, there is a relative dearth of information concerning the use of this approach in the eye. Studies have demonstrated the beneficial effects of MSC-derived exosomes in the treatment of autoimmune uveitis cells [54,55,56,57], retinal injury, and refractory macular holes. Regarding pathologies of the ocular surface, two studies have shown that MSC-Exo improves corneal wound healing and reduces opacity in a model of mucopolysaccharidosis [58,59]. However, further work is needed to understand the therapeutic efficacy of MSC-derived exosomes on ocular surface diseases. In this present study, we aimed to shed light on whether MSC-EV/Exo mediates the wound-healing effects of MSC-S in the cornea.

## 2. Materials and Methods

### 2.1. Cell Culture

A human corneal epithelial cell (HCEC) was cultured in 10% fetal bovine serum (FBS; #F2442, Sigma-Aldrich, St. Lois, MO, USA), 1X L-glutamine (#MT25005CI, Corning, Corning, NY, USA), 1X NEAA (#11140050, Gibco, Bilings, MT, USA), and 1% penicillin–streptomycin (P/S; #MT30002CI, Corning, Corning, NY, USA) in 5% CO_2_ at 37 °C [3]. A human corneal limbal epithelial (HCLE) cell line was cultured in 1% P/S and keratinocyte serum-free medium (KSFM; #17005-042, Thermo, Waltham, MA, USA) with 0.05 mg/mL of Bovine Pituitary Extract and 5 ng/mL of Epidermal Growth Factor [3]. Human bone marrow (BM)-derived MSCs were purchased from RoosterBio (# MSC-CC040, Ballenger Creek, MD, USA) and cultured in MEM alpha (MEMa; #4106102, ThermoFisher Scientific, Waltham, MA, USA) media supplemented with 10% FBS, 1X L-glutamine, and 1X NEAA [3,60].

### 2.2. Cell Viability

HCEC 3 × 10^5^ cells/well with 6 wells per group were cultured in the presence of various media (MEMa, MSC-CM, and MSC-CM without exosome) for 24 h. The cells were rinsed with 1X PBS (#10-010-023, Gibco, Bilings, MT, USA) twice, trypsinized, and then incubated with trypan blue (TB; # T4049, Sigma-Aldrich, St. Lois, MO, USA) for 10 min at room temperature. A total of 20 μL of TB was mixed with 20 μL of cell supernatant and examined immediately under a microscope at 10× magnification. The number of blue-stained cells and the number of total cells were counted to determine the number of live and dead cells, and to the calculate cell viability: % of Viable cells = [1.00 − (Number of blue cells ÷ Number of total cells)] × 100. These analyses were performed in triplicate (*n* = 3) [3].

### 2.3. Cell Proliferation

HCEC and HCLE cells were plated on a 4-well chamber slide for 12 h. After incubation, the cells were washed twice with 1X PBS and cultured using a different medium (MEMa, MSC-CM, and MSC-CM without exosome) for 24 h. Cell proliferation was assessed by measuring the cellular DNA content via a fluorescent dye binding kit (CyQuant^®^ NF Cell Proliferation Assay; #C35006, Invitrogen, Waltham, MA, USA). After 24 h of incubation, the supernatant was removed with a pipette, and 100 μL of 1X CyQuant dye binding solution was added to each well. The plates were then incubated at 37 °C for 1 h. The fluorescence intensity for these samples was measured with an excitation wavelength of 485 nm and an emission wavelength of 530 nm using a Gen5 plate reader. All analyses were performed in triplicate (*n* = 3) [3].

### 2.4. LDH Toxicity Assay

For the LDH cytotoxicity assay, the cells were seeded on 96-well plates at a concentration of 3 × 104 cells per well. After 12 h of incubation, the cells were cultured in the presence of various media (MEMa, MSC-CM, and MSC-CM without exosome) and LDH positive for 24 h. After 24 h of incubation, 50 μL of supernatant was mixed with 50 μL of reaction mix according to the manufacturer’s instructions (#C2030, ThermoFisher Scientific, Waltham, MA, USA) in a 96-well flat-bottom plate and incubated at room temperature for 30 min. Optical density absorbance at wavelengths (490–680 nm) was measured using a Cytation5 (Biotek, Winooski, VT, USA) plate reader. These analyses were performed in triplicate (*n* = 3) [3].

### 2.5. In Vitro Scratch Assay

HCLE cells were seeded on 6-well plates at a concentration of 0.5 × 10^6^ cells per well, in KSFM complete with growth supplements. After 12 h of incubation, cell monolayers were scratched using a sterile 200 μL pipette tip and washed twice with 1X PBS to remove floating cells. Then, the treatments were added to the cells. After each time point, the scratch area was captured serially using a spinning disc confocal microscope (Z1; Carl Zeiss Meditec, Jena, Germany), and the remaining wound area was measured using ImageJ software (National Institutes of Health, Bethesda, MD, USA). These analyses were performed in triplicate (*n* = 3) [3].

### 2.6. Mouse Model of Corneal Epithelial Wound Healing

To compare the effects of the duration of CM collection (48 h vs. 72 h) and to examine the effect of EV (“exosome”) depletion, a 2 mm diameter wound was made on the center of the cornea (Mil-Tec, Lehigh Acres, FL, USA) with a rotating burr (Algerbrush II, The Alger Companies, Lago Vista, TX, USA) under a microscope as we have described before [3]. After the injury to the epithelium, mice were divided into four groups: PBS control, MSC-CM 48 h, MSC 72 h, and MSC-CM 72 h (-exosome) (*n* = 5). To study MSC-CM in a more extensive injury model, we used a limbus-to-limbus epithelial debridement model. Two different dilutions of MSC-CM (0.5X and 1X) and exosome-depleted MSC-CM (1X) were applied to the murine cornea surface twice a day for 7 days. These analyses were performed in triplicate (*n* = 3). For all the experiments, 10 μL of the topical treatment was applied to the right eye of each mouse twice a day. Fluorescein (1 mg/mL, BioGlo; #NDC 17238-900-30) was applied to the cornea for 1 min and excess liquid was removed using Kimwipes. The fluorescein staining was examined and imaged with a slit lamp (Nikon FS-2) using 30× magnification. The fluorescein intensity ratio data analysis was processed using MetaMorph software (Molecular Devices, Version 7.8.13.0).

### 2.7. Histology

Mice eyes were collected from the in vivo experiments (limbus-to-limbus wound and 2 mm wound with treatment: PBS, 0.5X MSC-CM, 1X MSC-CM, and 1X MSC-CM without exosome, MSC-CM 48 h, MSC-CM 72 h, and MSC-CM 72 h without exosome). At the end of the experiment, the mice eyes were collected and embedded in OCT medium. Cryo-sections at 10 μm were made and fixed in neutral buffered 10% formaldehyde (PFA; #F1635, Sigma-Aldrich, St. Louis, MI, USA) for 20 min and followed by Hematoxylin and Eosin staining per the protocol as previously described [3].

### 2.8. MSC Conditioned Medium (Secretome) and Storage Conditions

To produce MSC-conditioned media, human BM-MSCs were seeded at a density of 10,000 cells/cm^2^ in T75 flasks for 48 h for monolayer confluency of about 70–80% under the microscope. If the cells did not reach 70–80% confluence, they were incubated for an additional 12–24 h. Once the cells were ready, the medium was removed and gently washed twice with 10 mL of 1X DPBS. Then, the cells were incubated with a MEMa for an additional 72 h. After the incubation period, the media were collected and centrifuged at 500× *g* for 100 min to remove cells or debris. The supernatant from the centrifuge tubes was carefully removed leaving 1 mL of supernatant at the bottom of the centrifuge tubes to exclude possible cell/debris contamination. The collected CM was refrigerated right away at −80 °C. To deplete the exosomes, the collected medium was ultracentrifuged (100,000× *g* for 4 h); then, the supernatant was carefully removed without touching the bottom. To obtain freeze–thawing-conditioned media, the BM-MSC-conditioned media were stored at −80 °C for long-time storage. For non freezing MSC-CM, MSC-CM was stored at 4 °C for 1 week without freezing. For freeze–thawing MSC-CM, MSC-CM was thawed on ice and kept at 4 °C for 1 day, 1 week, and 4 weeks. For control media, the MEMa basal media were stored in cell-free T75 flasks in a cell culture incubator at 37 °C for 72 h.

### 2.9. Nanoparticle Tracking Analysis (NTA)

Particle size distribution in MSC-CM was determined by NTA using a NanoSight NS300 system (Malvern Technologies, Malvern, UK) [61]. Samples were diluted in particle-free 1X PBS (1:20–1:50) to a final volume of 1 mL to measure the ideal particle per frame value (20–100 particles/frame). A total of 1 mL of CM in PBS was transferred to a 1 mL Luer syringes (NORM-JECT) without air bubbles and was inserted into the NTA chamber. The particles scattering light are detected by a charge-coupled device and recorded in multiple video frames for a limited time to measure the size and density of the samples. These analyses were performed in triplicate (*n* = 3).

### 2.10. Medium Preparation and Endotoxin Quantification

MSC-CM was collected under different conditions, then ultracentrifuged (100,000× *g* for 4 h) to deplete exosomes. A total of 18 samples of MSC-CM from the 3 different productions were stored at −80 °C before the endotoxin assay. The endotoxin content in MSC-CM was determined quantitatively using a Pierce Chromogenic Endotoxin Quant Kit (#A39552, Thermo Fisher Scientific, Waltham, MA, USA) as per the manufacturer’s protocol. The endotoxin standards, samples, and blanks were all run in triplicate. Samples were pre-equilibrated in a 37 °C water bath. A total of 50 μL of endotoxin standard dilution, blank, and samples per well were used to measure the endotoxin level. Optical density (OD) at 405 nm was read immediately after assay completion using a Gen5 plate reader.

### 2.11. Bacterial Growth

Staphylococcus aureus ATCC 29213 was grown in Tryptic Soy Agar (TSA; #236950, Difco, St Louis, MO, USA). A total of 40 g of TSA powder was dissolved in 1 L of purified water, mixed well, sterilized using autoclaving at 121 °C for 15 min, and cooled to 45–50 °C. The agar was thoroughly mixed well and poured into Petri dishes. To prepare the bacterial growth, 20 μL of −20 °C glycerol stock and three different MSC-CM were spread out on TSA plates and cultured at 37 °C for 3 days. The bacterial growth of the MSC-CM spread plate was compared to the positive control plate and captured on day 3.

### 2.12. Statistical Analysis

Statistical calculations were conducted using GraphPad Prism software (GraphPad, La Jolla, CA, USA). The results are presented as the mean ± standard deviation (SD) of three independent experiments. *p*-values for the differences were determined using double-sided nonparametric *t*-tests, with GraphPad Prism software and Microsoft Excel. A *p*-value of <0.05 was considered statistically significant.

## 3. Results

### 3.1. MSC-CM Promotes Cell Proliferation Which Is Diminished in EV-Depleted MSC-CM

**Figure 1:**
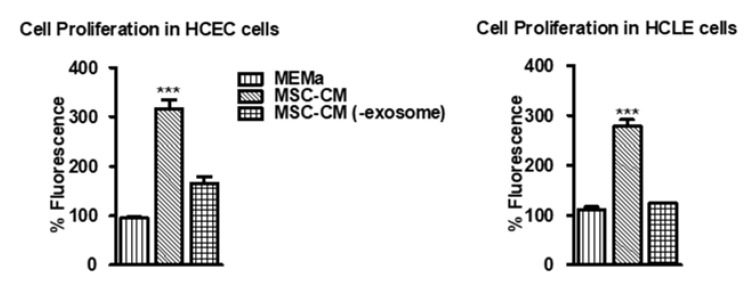
MSC-CM promotes cell proliferation which is diminished in EV-depleted MSC-CM. Graph showing cell proliferation in culture cells (HCEC and HCLE) as measured (*n* = 6/group). *** *p* < 0.001 vs. MEMa group.

The effect of MSC-CM and EV-depleted MSC-CM on viability, cytotoxicity, and cell proliferation was measured in human corneal epithelial cells (HCEC) and a human corneal limbal epithelial (HCLE). Cell viability at 24 h was not significantly different between any of the incubated media (Figure S1A). As another more sensitive measure of toxicity, a lactate dehydrogenase (LDH)-based assay was used to measure LDH in HCEC exposed to the MSC-CM and EV-depleted CM and MEMa and did not show any significant difference (Figure S1B). Ultracentrifugation at 100,000× *g* for 4 h resulted in a >80% reduction of exosomes in MSC-CM (Figure S1C). There was no difference in endotoxin levels between MSC-CM and exosome-depleted MSC-CM (Figure S1D). Additionally, MSC-CM was free of bacterial contamination (Figure S1E). MSC-CM did significantly increase cell proliferation in HCEC and HCLE cells compared to MEMa (Figure 1). In contrast, EV-depleted MSC-CM showed lower cell proliferation in HCEC and HCLE cells compared to MSC-CM (for HCEC cells, MEMa: 86.87 ± 22.19%, MSC-CM: 316.68 ± 44.75%, and MSC-CM (-exosome): 166.18 ± 32.43%, *** *p* < 0.001; for HCLE cells, MEMa: 111.46 ± 13.42%, MSC-CM: 278.22 ± 31.65%, and MSC-CM (-exosome): 123.79 ± 3.19%, *** *p* < 0.001).

### 3.2. MSC-CM Dose-Dependently Promotes Wound Healing In Vitro Which Is Dependent on the EV Fraction

**Figure 2:**
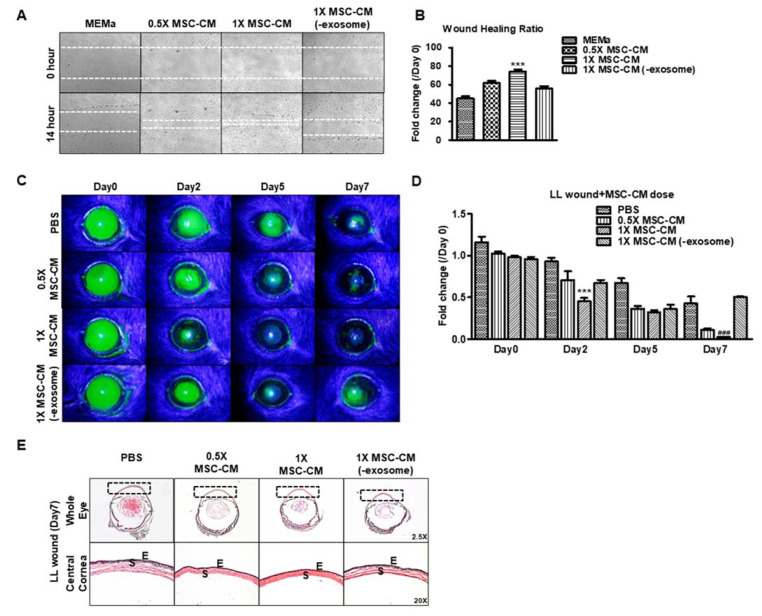
MSC-CM dose-dependently promotes wound healing in vitro which is dependent on the EV fraction: (**A**) Representative images showing scratch wound assay in HCLE cells. White dot: wound area. (**B**) Graph showing wound healing rate for different conditions in epithelial scratch wounds (*n* = 5/group) at 14 h. *** *p* < 0.001 vs. MEMa, 0.5X MSC-CM, or 1X MSC-CM (-exosome). (**C**) Representative images of murine corneas showing fluorescein staining after limbus-to-limbus wound and application of PBS, 0.5X MSC-CM, 1X MSC-CM, and 1X MSC-CM (-exosome). 30X. (**D**) Graph showing the intensity fold change of corneal fluorescein staining after application of PBS, 0.5X MSC-CM, 1X MSC-CM, and 1X MSC-CM (-exosome) for 48 h (*n* = 4), *** *p* < 0.001; vs. 0.5X MSC-CM or 1X MSC-CM (-exosome) on Day 2, ^###^ *p* < 0.001; vs. 0.5X MSC-CM or 1X MSC-CM (-exosome) on Day 7. LL: limbus-to-limbus. (**E**) H&E staining on whole eyeball after application of PBS and MSC-CM for 7 days. Black box: central cornea, E: epithelium, S: stroma.

We used an in vitro scratch assay to determine whether the EV component of MSC-CM contributes to its wound-healing effect. Human MSC-CM was collected and used in the following experimental conditions: (i) 0.5X MSC-CM (MSC-CM diluted 1:1 with MEMa); (ii) 1X MSC-CM; (iii) 1X MSC-CM (-exosome = EV-depleted). A concentration of 1X MSC-CM significantly enhanced wound healing compared to 0.5X MSC-CM and 1X MSC-CM (-exosome) at 14 h (61.8 ± 4.57% vs. 74.9 ± 7.65% vs. 55.7 ± 6.10%) (Figure 2A,B). The 1X MSC-CM treatment showed significantly less fluorescein staining (0.45 ± 0.08) compared to other groups (PBS: 0.93 ± 0.09, 0.5X MSC-CM: 0.71 ± 0.20, and 1X MSC-CM (-exosome): 0.67 ± 0.08) on day 2. On the 7th and final day, 1X MSC-CM showed significantly less staining than 0.5X MSC-CM (0.02 ± 0.006 vs. 0.11 ± 0.02), whereas EV-depletion delayed wound healing compared to other groups (PBS: 0.32 ± 0.11, 0.5X MSC-CM: 0.11 ± 0.02, 1X MSC-CM: 0.02 ± 0.06, and 1X MSC-CM (-exosome): 0.5 ± 0.007) (Figure 2C,D). The H&E-stained cross-section of cornea exhibited an LL wound that was completely healed and the corneal stromal was tightly packed after 7 days of treatment with 0.5X or 1X MSC-CM and showed normal morphology of the epithelial and stroma (Figure 2E). However, the PBS or 1X MSC-CM (-exosome) groups showed that the corneal stroma was more swollen compared to the 0.5X or 1X MSC-CM groups (Figure 2E). Taken together, MSC-CM promotes wound healing in a dose-dependent manner.

### 3.3. MSC-CM Promotes Wound Healing In Vivo Which Is Dependent on the EV Fraction

We studied the effect of MSC-CM after a 2 mm epithelial wound in vivo. At 24 h after wounding, the corneas treated with BM-MSC-conditioned media healed significantly faster (*p* < 0.001, 0.1 ± 0.04% vs. 0.3 ± 0.05). By 48 h all wounds had healed (Figure S3). No adverse effects were noted on the exam. Microscopic analysis of murine eyes showed that an H&E-stained cross-section of cornea exhibited a 2 mm wound that was completely healed after 7 days of treatment and showed normal morphology of the epithelial and stroma (Figure S2). Taken together, MSC-CM applied twice a day topically accelerates corneal epithelial wound healing in vivo.

**Figure 3:**
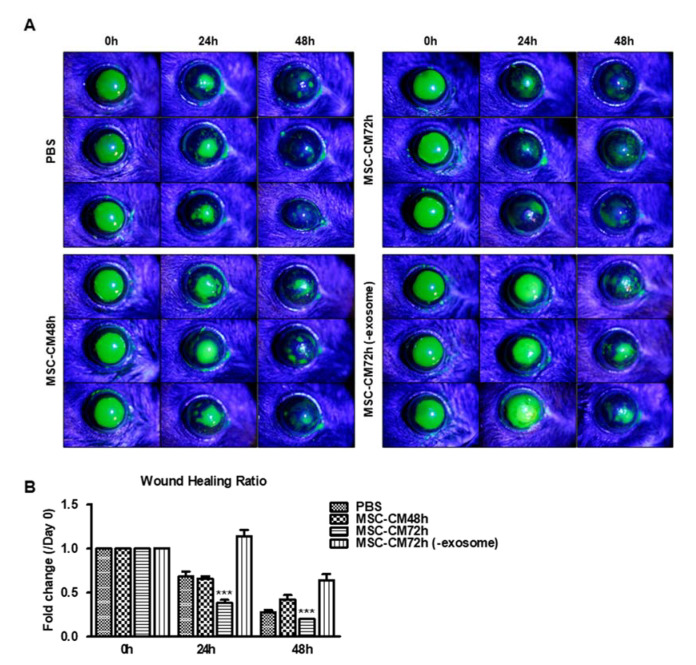
MSC-CM promotes wound healing in vivo which is dependent on the EV fraction: (**A**) Representative images of murine corneas showing fluorescein staining after a 2 mm wound and application of PBS, MSC-CM 48 h, MSC-CM 72 h, and MSC-CM 72 h (-exosome) for 48 h. 30X. (**B**) Graph showing the intensity fold change of corneal fluorescein staining after application of PBS, MSC-CM 48 h, MSC-CM 72 h, and MSC-CM 72 h (-exosome) for 48 h (*n* = 5); *** *p* < 0.001 vs. MSC-CM 48 h or MSC-CM 72 h (-exosome) at 24 and 48 h.

MSC-CM 72 h decreased the ratio of the fluorescein intensity as compared to the PBS control group at 24 h (0.39 ± 0.10 vs. 0.68 ± 0.13). MSC-CM 48 h had reduced cornea wound-healing effects compared to MSC-CM 72 h (0.68 ± 0.06 vs. 0.39 ± 0.10). EV-depleted MSC-CM 72 h (-exosome) delayed corneal wound healing as compared to the MSC-CM 72 h and PBS control groups (1.12 ± 0.17 vs. 0.39 ± 0.10 vs. 0.68 ± 0.13) (Figure 3). The results confirm the in vitro data that the EV fraction in MSC-CM has a critical role in the cornea wound-healing effects.

Collectively, MSC-CM promoted in vivo wound healing in a dose-dependent manner and the depletion of EV/exosome from MSC-CM significantly reduced these effects. The findings suggest that EV/exosomes in MSC-CM are one of the key factors that mediate the cornea wound-healing effects.

### 3.4. The Effects of Storage Conditions on the Wound-Healing Effects of MSC-CM

**Figure 4:**
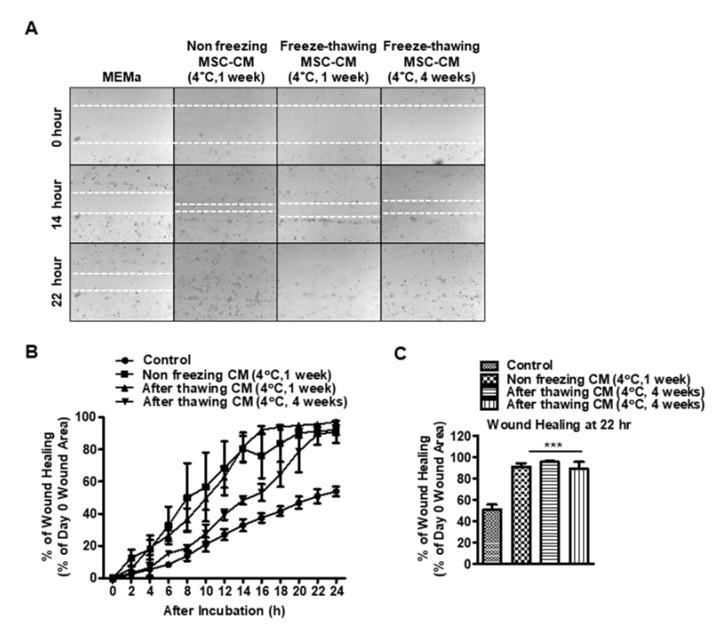
The effects of freeze–thawing MSC-CM on wound healing: (**A**) Representative images showing scratch wound assay in HCLE cells that are incubated with various conditions; (i) MEMa, (ii) Non freezing MSC-CM (stored at 4 °C for 1 week without freezing), (iii) Freeze–thawing MSC-CM (stored at 4 °C for 1 week after thawing), and (iv) After thawing MSC-CM (stored at 4 °C for 4 weeks after thawing). White dot: wound area. (**B**) Kinetic curve showing the relative wound healing at different time points. (**C**) Graph showing wound healing rate for different conditions in epithelial scratch wounds (*n* = 5/group) at 22 h. *** *p* < 0.001 vs. freeze–thawing MEMa. Control: MEMa.

Human MSC-CM was collected and stored under various conditions to determine the stability of the wound-healing effects. We wished to examine whether freeze–thawing had any effect and also whether it remained stable at 4 °C after thawing. As shown in Figure 4, MSC-CM stored at 4 °C (no freezing) for 1 week promoted wound healing (91.33 ± 2.82% healed at 22 h) which was comparable to MSC-CM that had been frozen then thawed and stored at 4 °C for 1 week (95.57 ± 0.86% healing at 22 h). Freeze–thawed MSC-CM stored at 4 °C for 4 weeks did show some signs of delayed wound healing compared to non-frozen MSC-CM at 14 h, but at 22 h it did reach 89.4 ± 6.3% healing. Finally, reconstituted CM after freeze–thawing showed significantly faster healing compared to fresh MSC-CM.

## 4. Discussion

Our previous study investigated the use of a novel therapy for corneal wound healing using a conditioned medium derived from corneal mesenchymal stromal cells [62]. In addition, we explored the use of mesenchymal stem cell secretome delivered within a viscoelastic gel carrier as a potential therapy for corneal wound healing [63]. Both studies demonstrate promising results and highlight the potential of mesenchymal stromal cells and their secreted factors as a therapeutic option for treating corneal epithelial wounds. This study investigated the use of human bone-marrow-derived MSC-CM for corneal epithelial wound healing. We show that MSC-CM promotes epithelial cell migration and proliferation, resulting in corneal wound-healing effects. Previous studies have identified that MSC-CM or MSC-EV/Exo can both enhance wound healing in vitro and in vivo [3,36,64,65,66]. We have also previously demonstrated that human corneal MSC-derived EV/exosomes promote the repair of ocular surface injuries [62,67,68]. Current findings show that the EV/Exo fraction mediates the wound-healing effects of human bone-marrow MSC-CM in the cornea and importantly show that the depletion of EV/Exo from MSC-CM significantly abolishes the corneal wound-healing effects. These effects were observed both in vitro and in vivo in this study. It is becoming increasingly clear that MSCs do not exert their beneficial effects by migrating to the target site and differentiating into functional replacement cells. Rather, their paracrine release of EV/Exo laden with cytokines, peptides, RNA, and other bioactive factors appears to drive the positive effects on wound healing [69]. Our study, therefore, adds to the growing body of evidence that topical MSC-EV/Exo can be an effective method to improve wound healing of the ocular surface. Further studies are needed to determine whether the efficacy of topical MSC-EV/Exo therapy holds for other ocular surface disorders as well.

Limbal stem cells are crucial in the regeneration of the corneal epithelium and in maintaining corneal transparency and visual acuity [70]. Impaired healing of the corneal epithelium and conjunctivalization of the corneal surface can result from genetic defects or damage to the LSCs and their niche, leading to LSCD [71,72]. Pellegrini et al. demonstrated that using autologous limbal stem cells can effectively treat corneal damage due to burns and the limbal stem cell deficiency (LSCD) [70,73]. Mesenchymal stem-cell-derived extracellular vesicles (MSC-EV) could also provide a potential treatment option due to their pro-regenerative, anti-inflammatory, and anti-fibrotic effects, which make them a safer alternative to limbal stem cell transplantation. Moreover, EVs are easily purified and isolated, making them a more convenient option. Although limbal stem cells are still the gold standard for treating LSCD, the potential of MSC-EVs in cases of major corneal defects is an exciting area of research. Further studies are needed to fully understand the mechanisms underlying the therapeutic effects of MSC-EVs and to determine the optimal approach for their clinical application.

MSC therapy refers to the direct administration of MSCs to patients for therapeutic purposes, with regulatory aspects focused on ensuring their safety and efficacy [74,75]. On the other hand, MSC-derived products are produced by isolating and expanding MSCs in vitro, and using the resulting, conditioned media, or extracellular vesicles as therapeutic products [76]. Regulatory aspects for an MSC-based product include ensuring the safety and purity of the final product, establishing its identity and potency, and ensuring consistency in production [74,77]. However, the lack of standardization in the MSC field poses a significant regulatory challenge for MSC-based products [78], due to the heterogeneity of MSCs and the difficulty in establishing consistent manufacturing processes that result in products with consistent quality attributes [79,80]. This can lead to variability in the safety and efficacy of MSC-based products, making it difficult for regulatory agencies to evaluate and approve them for clinical use. Establishing consistent standards for the characterization and testing of MSC-based products will be important for ensuring their safety and efficacy in clinical applications [81]. Further research is also necessary to identify all the active biomolecules in MSC-based products and understand their mechanisms of action [31,55,82].

The advantages of using MSC-S for corneal wound healing are that MSC-S contains a variety of growth factors, cytokines, and extracellular matrix proteins that can promote wound healing [18,19,20,21]. The use of MSC-S can also potentially overcome the limitations of traditional cell-based therapies, such as the need to maintain cell viability and concerns about immunogenicity [9]. Additionally, the use of MSC-S may provide a more standardized and reproducible approach compared to traditional cell-based therapies. Another advantage of using MSC-EV/Exos is that they can be easily isolated from MSC-S and stored for future use. We demonstrate that MSC-S is stable under different storage conditions (Figure 4), and this provides a measure to optimize its dosing for a potential clinical product (Figure 2). The findings of this study provide important insights into the use of MSC-EV/Exo as a therapeutic agent for corneal wound healing. The immunomodulatory and regenerative properties of MSC-EV/Exos make them an ideal candidate for the treatment of corneal wounds, and their ease of isolation and long-term stability make them a promising approach for the development of corneal therapies.

These findings support the transition of cell-based MSC therapy to more targeted approaches using conditioned media and exosomes. Exosomes can be administered via multiple routes: intravenously, intramuscularly, subcutaneously, intrathecally, and even topically with a spray [83]. Their efficacy in preclinical studies coupled with their flexibility of delivery have made exosomes a topic of interest for researchers in both universities and private companies. Currently, multiple start-up companies are researching the translational potential of exosome therapy [83]. While more than 100 clinical trials on exosomes are ongoing, only 28% study them as a therapeutic modality [84]. Here too, further clinical trials are needed to translate the results of these preclinical studies into effective therapies for patients. Further clinical trials are needed to evaluate the safety and efficacy of using MSC-conditioned media to treat corneal epithelial wounds in humans. These trials should include a larger patient population and should assess various parameters such as the quality of conditioned media, optimal dosage (0.5X or 1X as shown in Figure 2), frequency and duration of treatment, as well as potential adverse effects. In addition, further research is needed to elucidate the mechanisms underlying the therapeutic effects of MSC-conditioned media and to optimize its production and storage (as shown in Figure 4). This could involve exploring the use of different types of MSCs or different methods for isolating and purifying MSC-derived extracellular vesicles. Overall, while the results of preclinical studies are promising, it is important to conduct rigorous clinical trials to ensure the safety and efficacy of MSC-conditioned media before it can be used as a therapy for corneal epithelial wounds.

We also compared the therapeutic effects of CM collected over 48 or 72 h on wound healing. As shown in Figure 3, CM collected over 72 h promoted wound healing to a greater extent than CM from 48 h of incubation. Similarly, we investigated whether the observed effects of MSC-CM are dose-dependent. Diluted-conditioned media to 0.5X (in MEMa) revealed that this still has healing capacity to enhance healing compared to the control but less than non-diluted 1X CM (Figure 2). As mentioned, EV/exosome depletion significantly diminished the wound-healing effects. Overall, these results show that there is a dose-dependent effect and that collecting a conditioned medium over 72 h may be advantageous over 48 h.

Preservation of exosomes is also an area of active investigation [85,86]. Freeze–thawing and storage temperature may change the EVs and decrease their biological activity [87,88,89,90]. The Bosch et al. group demonstrated that the addition of 25 mM of trehalose prevents the aggregation of exosomes and cryodamage, resulting in the improved preservation of biological activity [85]. Here, we present findings on the stability of CM for up to 4 weeks and show that the storage condition does not have a significant impact on the stability and therapeutic functions. As shown in Figure 4, different storage conditions (non-freezing, one-time freezing, and storage for 1 day, 2 days, 1 week, and 4 weeks) revealed that there is no loss of biofunction up to 4 weeks of storage at 4 degrees compared to MSC-CM that was not frozen. Previously, we demonstrated that lyophilized corneal MSC-CM is effective for the promotion of wound healing and modulation of inflammation in both in vivo and in vitro models [62].

In summary, our study has shed light on the mechanisms underlying the wound-healing effects of MSC-CM in the corneal epithelium. By demonstrating that EV/exosomes are the active ingredient responsible for these effects, we have provided a potential strategy for standardizing and optimizing the dosing of MSC-CM for clinical use. This is a significant step forward in the development of MSC-CM as a therapeutic approach for corneal injuries and diseases. Furthermore, our findings highlight the importance of further investigating the cargo and active components within EV/exosomes that mediate these effects, to improve the therapeutic potential of MSC-CM. The improved corneal barrier function and decreased corneal haze/edema observed with EV/Exo-containing MSC-CM treatment further emphasizes the therapeutic potential of this approach. Finally, the long-term stability of MSC-CM demonstrated in this study provides additional support for its potential clinical translation. Taken together, our findings provide important insights into the potential of MSC-CM and EV/exosomes as a novel approach for the treatment of corneal injuries and diseases. Further research is needed to fully realize this potential and advance this promising field of study.

## 5. Conclusions

MSC-Exos is the active ingredient in MSC-CM that promotes epithelial cell migration and proliferation, resulting in wound-healing effects in the corneal epithelium. This is important given that it can be used to standardize and determine the dosage of MSC-CM. In other words, we propose that MSC-CM can be dosed based on the EV/Exo concentration. Further studies are needed to further characterize the cargo and the most active components within the EV/Exosomes that mediate the observed wound-healing effects.

## Data Availability

Any data or material that supports the findings of this study can be made available by the corresponding author upon request.

## Supplementary Materials

The following supporting information can be downloaded at: https://www.mdpi.com/article/10.3390/pharmaceutics15051486/s1, Figure S1: Cell viability (Trypan blue staining), nanoparticles, endotoxin, and bacterial culture; Figure S2: Human MSC-CM helps corneal wound healing; Figure S3. Effects of pre-processing storage condition of MSC-CM on corneal wound healing in vitro 19 scratch assay.
