# Supplementary material for: Wound-Healing Effects of Mesenchymal Stromal Cell Secretome in the Cornea and the Role of Exosomes
Source: Pharmaceutics. 2023 May 13;15(5):1486. doi: 10.3390/pharmaceutics15051486 (PMC10221647; doi:10.3390/pharmaceutics15051486)
Supplement: Supplementary file 1 [file pharmaceutics-15-01486-s001.zip › pharmaceutics-2355213-supplementary.pdf]

pharmaceutics

MDPI

Article

Wound Healing Effects of Mesenchymal Stromal Cell Secretome in the Cornea and the Role Exosomes

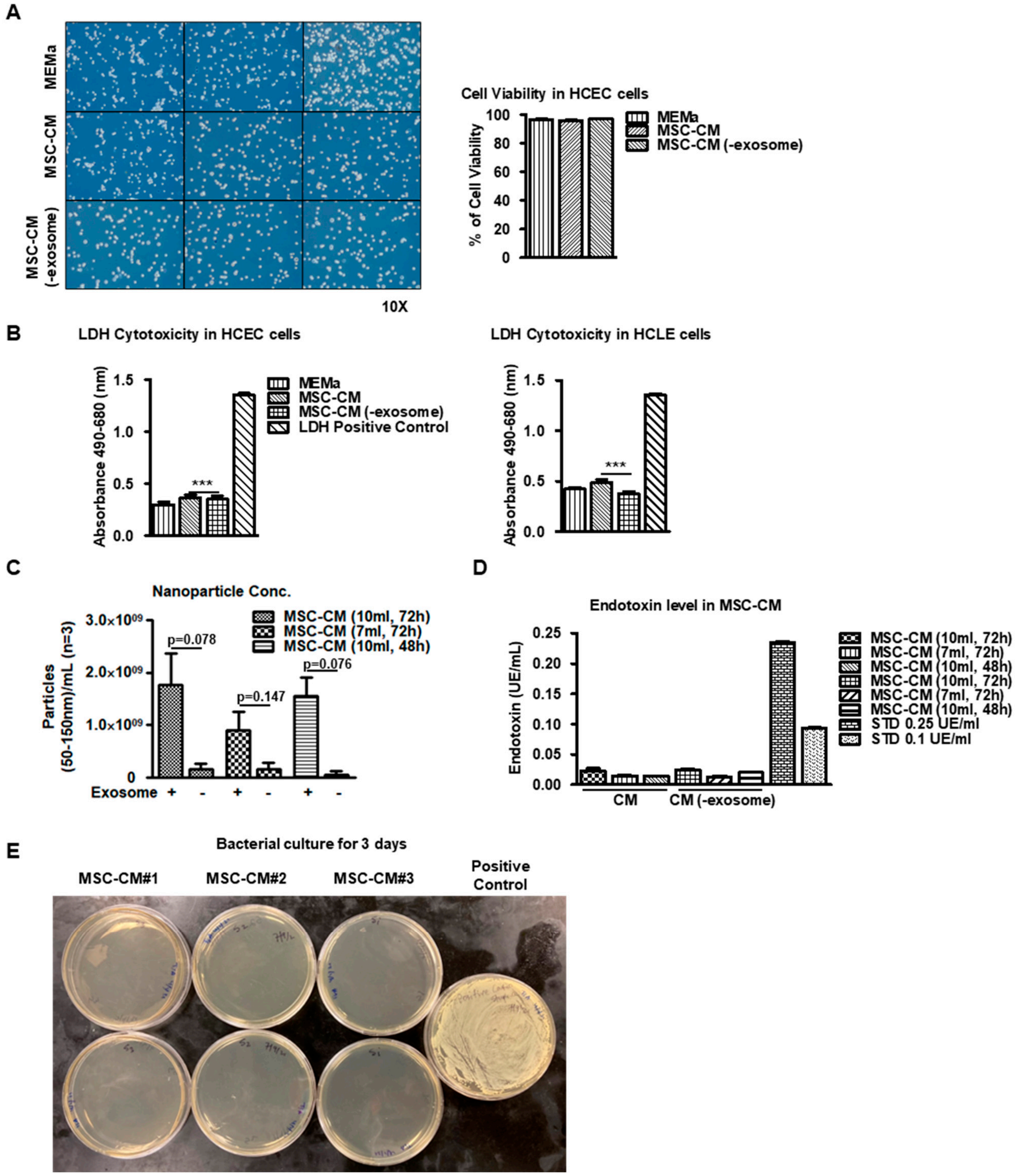

Supplementary Figure S1. Cell viability (Trypan blue staining), nanoparticles, endotoxin, and bacterial culture. (A) Cell viability was measured after incubation with a different medium for 24 h in HCEC cells (n=10/group). (B) Graph showing

cytotoxicity for various media in culture cells (HCEC and HCLE) as measured by LDH assay (n=6/group). *** $p < 0.001$ vs. STD groups (0.25 and 0.1 UE/ml). (C) Human MSC cells were incubated in different conditions to generate CM for 72 h. Nanoparticles were measured by NanoSight (n=3/group). (MSC-CM 10ml,72h: p=0.039, MSC-CM (7ml, 72h): p=0.147, MSC-CM (10ml, 48h): p=0.07). -: exosome depleted MSC-CM. (D) Graph showing endotoxin levels in MSC-CM with or without exosome (n=3/group). There was no difference in endotoxin levels between MSC-CM and exosome-depleted MSC-CM. (E) MSC-CM from different batches were used to check bacterial contamination (n=2/group). MSC-CM was free of bacterial contamination.

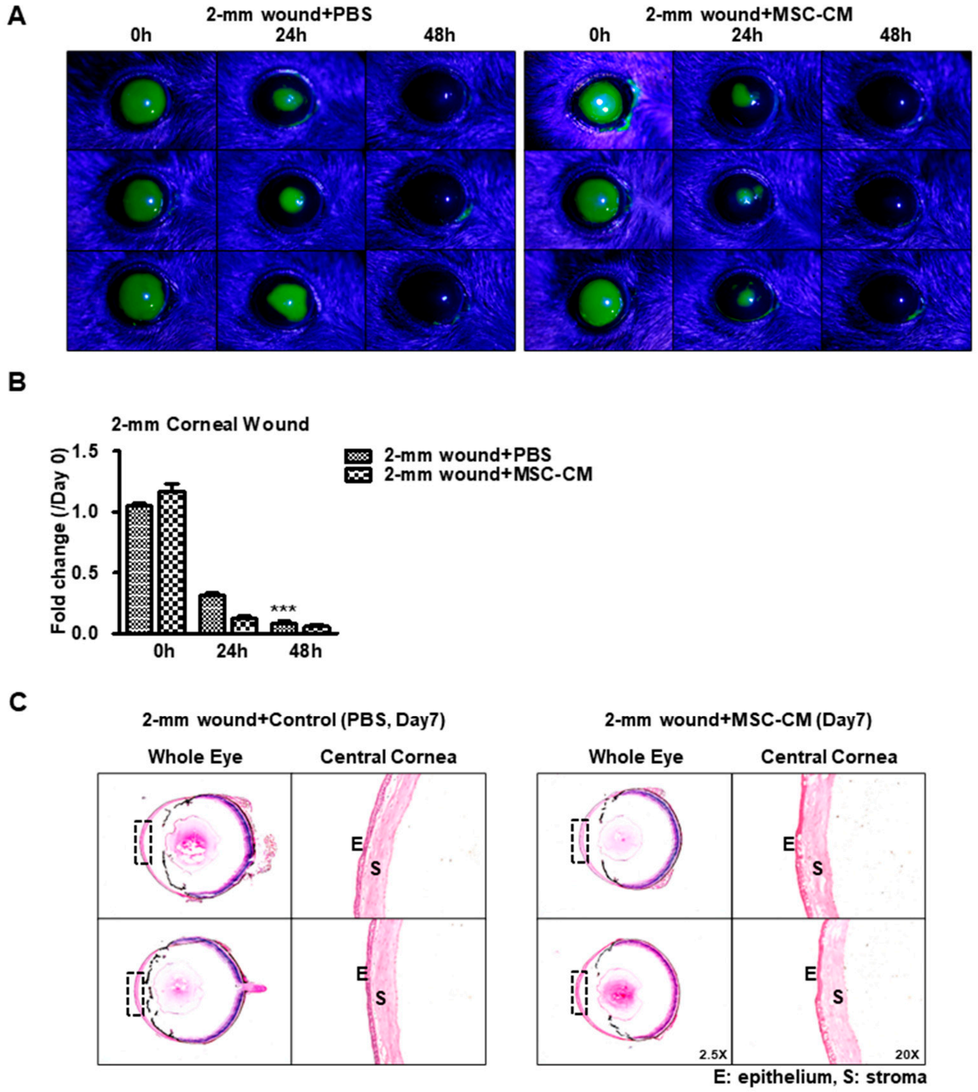

Supplementary Figure S2. Human MSC-CM helps corneal wound healing. (A) Representative images of murine corneas showing fluorescein staining after a 2-mm wound and application of PBS and MSC-CM for 48 h. (B) Graph showing the intensity of corneal fluorescein staining after application of PBS and MSC-CM for 48 h (PBS, n = 6, MSC-CM, n=7). *** $p < 0.001$ vs. 2-mm+PBS. (C) H&E staining on whole eyeball after application of PBS and MSC-CM for 7 days. Black box: central cornea, E: epithelium, S: stroma. Black box: central cornea.

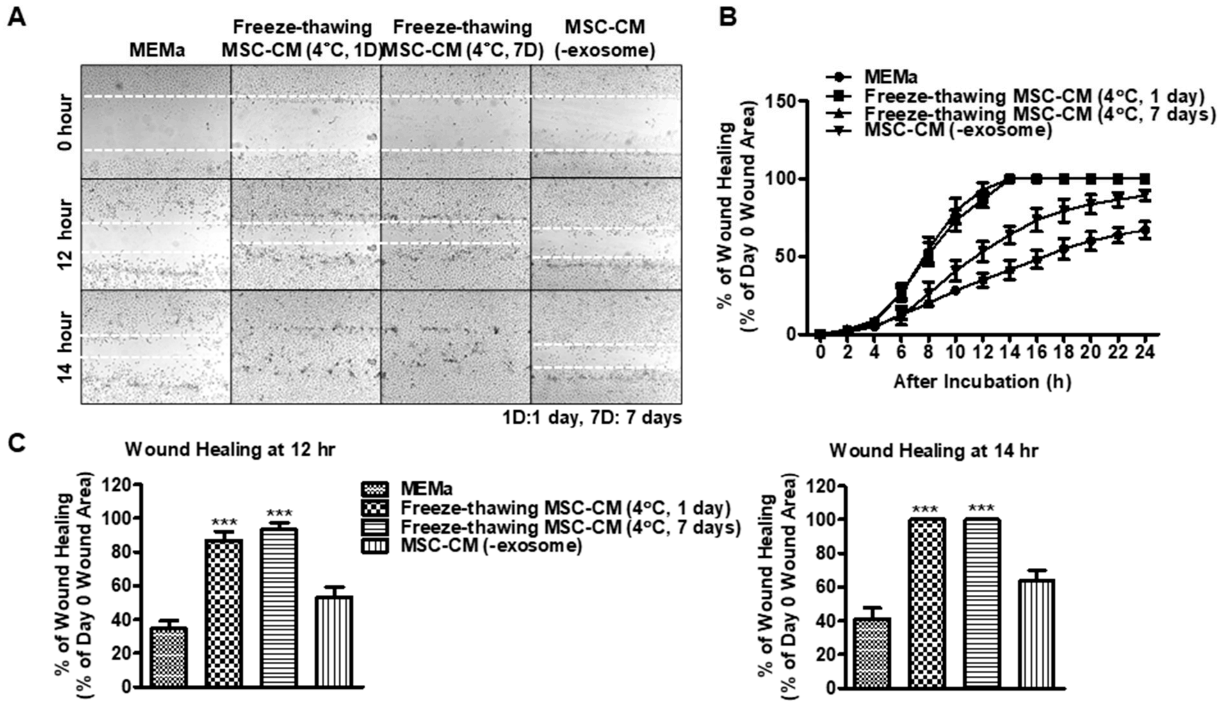

Supplementary Figure S3. Effects of pre-processing storage condition of MSC-CM on corneal wound healing in vitro scratch assay. (A) Representative images showing scratch wound assay in human corneal epithelial cells. White dot: wound area. (B) A kinetic curve showing the relative wound healing at different time points. (C) Graph showing wound healing rate for different conditions in epithelial scratch wounds (n = 5/group) at 12 or 14 h. *** $p < 0.001$ vs. MEMa or MSC-CM (-exosome).
